# RT-LAMP CRISPR-Cas12/13-Based SARS-CoV-2 Detection Methods

**DOI:** 10.3390/diagnostics11091646

**Published:** 2021-09-08

**Authors:** Kasturi Selvam, Mohamad Ahmad Najib, Muhammad Fazli Khalid, Suharni Mohamad, Fahreddin Palaz, Mehmet Ozsoz, Ismail Aziah

**Affiliations:** 1 Institute for Research in Molecular Medicine (INFORMM), Health Campus, Universiti Sains Malaysia, Kubang Kerian 16150, Kelantan, Malaysia; kasturiselvam0612@gmail.com (K.S.); najib@student.usm.my (M.A.N.); fazlikhalid@usm.my (M.F.K.); 2 School of Dental Sciences, Health Campus, Universiti Sains Malaysia, Kubang Kerian 16150, Kelantan, Malaysia; suharni@usm.my; 3 Faculty of Medicine, Hacettepe University, Ankara 06100, Turkey; fahreddinpalaz@hacettepe.edu.tr; 4 Department of Biomedical Engineering, Near East University, Nicosia 99138, Turkey

**Keywords:** COVID-19, SARS-CoV-2, RT-LAMP, CRISPR, Cas12, Cas13

## Abstract

Coronavirus disease 2019 (COVID-19), which is caused by severe acute respiratory syndrome coronavirus-2 (SARS-CoV-2), has attracted public attention. The gold standard for diagnosing COVID-19 is reverse transcription–quantitative polymerase chain reaction (RT-qPCR). However, RT-qPCR can only be performed in centralized laboratories due to the requirement for advanced laboratory equipment and qualified workers. In the last decade, clustered regularly interspaced short palindromic repeats (CRISPR) technology has shown considerable promise in the development of rapid, highly sensitive, and specific molecular diagnostic methods that do not require complicated instrumentation. During the current COVID-19 pandemic, there has been growing interest in using CRISPR-based diagnostic techniques to develop rapid and accurate assays for detecting SARS-CoV-2. In this work, we review and summarize reverse-transcription loop-mediated isothermal amplification (RT-LAMP) CRISPR-based diagnostic techniques for detecting SARS-CoV-2.

## 1. Introduction

Severe acute respiratory syndrome coronavirus 2 (SARS-CoV-2), the causative agent for coronavirus disease 2019 (COVID-19), emerged in December 2019 in Wuhan, China, and caused a pandemic [1]. Coronaviruses are large, positive-stranded ribonucleic acid (RNA) viruses with genome sizes ranging from ~27 to ~32 kilobase pairs (kbp), which is the largest genome size for RNA viruses [2]. Individuals above the age of 70, as well as those with underlying comorbidities, are at a higher risk of developing severe COVID-19, which includes pneumonia and acute respiratory distress syndrome (ARDS). Severe COVID-19 patients require mechanical ventilators to improve their breathing. Many others experience mild-to-moderate symptoms, including fever, lethargy, dry cough, and dyspnea or have no symptoms at all [3]. Countries worldwide have enforced stringent public health regulations, such as mandatory isolations, physical distancing, and travel restrictions, to stop the virus from spreading [4]. However, battling the evolving SARS-CoV-2 requires early detection and massive screening, especially for those with clinical symptoms similar to SARS-CoV-2 infection. Over half of the human-to-human transmissions of SARS-CoV-2 occur from asymptomatic carriers. Therefore, the aggressive contact tracking and isolation of asymptomatic carriers have proven to be quite efficient in limiting the virus spread [4]. Thus, rapid and accurate diagnostic tests are essential for detecting SAR-CoV-2 to fight against the pandemic.

The COVID-19 pandemic can be monitored and managed more effectively if the virus is detected early. Reverse transcription–quantitative polymerase chain reaction (RT-qPCR) has been considered the gold standard method and is the most widely used detection approach for SARS-CoV-2 [5]. Even though RT-qPCR has high sensitivity and reliability in detection, it is not suited for large-scale point-of-care (POC) diagnostics because it requires highly skilled workers, expensive equipment, and a long reaction time (turnaround time, TAT: 2–4 h) [5,6,7]. In addition, COVID-19 diagnosis by RT-qPCR is more challenging in certain low-resource areas [8]. Clinical samples must be processed in a biosafety level 2 (BSL-2) laboratory with unidirectional airflow or a biosafety level 3 (BSL-3) facility for the isolation of SARS-CoV-2 RNA virus before performing the RT-qPCR test. Therefore, this technique is challenging to execute during emergency scenarios where hundreds of samples must be evaluated as soon as possible to assess treatment choices and control an outbreak. 

As a result, approaches that meet the ASSURED (Affordable, Sensitive, Specific, User-friendly, Rapid and Robust, Equipment-free, and Deliverable to end-users) criteria for POC diagnostics are urgently required to combat the COVID-19 pandemic. CRISPR-Cas (clustered regularly interspaced short palindromic repeats and CRISPR-associated proteins) systems are molecular immunity mechanisms that protect bacteria and archaea against invading nucleic acids, such as phages and conjugative plasmids [9,10], and these systems have been used as powerful tools for genome and transcriptome editing, gene therapy, and nucleic acid detection [11]. Effector proteins of CRISPR-Cas systems are targeted to DNA or RNA sequences under the guidance of a CRISPR-RNA (crRNA). Among the single effector Cas endonucleases, Cas13 and Cas12 perform indiscriminate RNA and single-stranded deoxyribonucleic acid (ssDNA) cleavage, respectively, when they are activated with the crRNA target sequences [12,13]. This feature has been harnessed for reporting the presence of a defined RNA or DNA sequence in a sample, giving rise to the concept of CRISPR-based diagnostics [13,14,15,16]. CRISPR technology is a highly specific approach for detecting nucleic acids rapidly and accurately. CRISPR-based diagnostic methods offer ultrasensitive, less expensive, and portable diagnostic tests for evaluating suspected COVID-19 cases to aid in the diagnosis of SARS-CoV-2 infection. CRISPR-based diagnostic methods take advantage of diverse isothermal amplification approaches such as loop-mediated isothermal amplification (LAMP) and recombinase polymerase amplification (RPA), which yield the highly specific and sensitive amplification of a few copies of the targeted nucleic acid in a short period of time at a constant temperature, obviating the need for thermocycling steps. Thus, they are preferred for POC diagnostics where low cost and ease of use are required [4].

CRISPR-based techniques for the detection of SARS-CoV-2 RNA have been developed by combining RT-RPA with CRISPR-mediated detection. However, this combined method has some limitations, including being performed in two separate reaction steps, requiring a long incubation period (120 min), the generation of weak signals for low template concentrations, and having a strong background signal due to the multiple enzymes in the RPA system [7]. Furthermore, there are difficulties in the supply chain for the RPA reagents that are available on the market and challenges in developing a rapid and one-pot RPA test for the sensitive detection of SARS-CoV-2 [17]. However, LAMP is highly specific and produces a large yield of amplicon in a short period of time, with reagents that are less expensive and more readily available. Therefore, this review focuses on the CRISPR-based detection of SARS-CoV-2 RNA using Cas12 and Cas13 nucleases integrated with reverse-transcription LAMP (RT-LAMP).

## 2. RT-LAMP CRISPR-Cas Workflow

### 2.1. Sample Collection and RNA Extraction

The first stage in RT-LAMP CRISPR-based SARS-CoV-2 nucleic acid detection is sample collection from patients. Upper respiratory samples (nasopharyngeal swabs (NPSs), nasal swabs, oropharyngeal swabs (OPSs), nasopharyngeal aspirates, and nasal aspirates) are the most common forms of SARS-CoV-2 samples, followed by saliva, bronchoalveolar lavage, and sputum. Although NPS remains the gold standard for diagnostic testing for SARS-CoV-2, saliva has been proposed as an alternate sample source for SARS-CoV-2 detection. Saliva collection does not need specific consumables (swabs) and personal protective equipment (gloves and facemasks), causes less patient discomfort (non-invasive), and lowers the exposure risk for health professionals (direct interaction) [17,18,19]. As shown in Table 1, the number of clinical samples tested in the studies related to RT-LAMP CRISPR-based SARS-CoV-2 detection ranged from 8 to 378 samples. Various viral RNA extraction protocols have been authorized by the Centers for Disease Control and Prevention-Emergency Use Authorization (CDC-EUA). These include the DIRECTZOL KIT (Zymo Research, CA, USA), Qiagen DSP Viral RNA Mini kit (Qiagen, Hilden, Germany), and PureLink™ Viral RNA Mini Kit (Thermo Fisher Scientific, Waltham, MA, USA). The RNA isolation processes usually consist of three stages: lysis, separation, and the elution of RNA. However, as the number of COVID-19 cases increases, RNA extraction kits, and consumables become scarce. Therefore, several groups have achieved simple viral RNA extraction by combining chemical- and heat-based methods. For example, after the addition of lysis solution, the incubation of swab samples at 42 °C for 20 min and 64 °C for 5 min enabled RT-LAMP and Cas12a-based SARS-CoV-2 detection [20]. Similarly, the treatment of saliva samples with TCEP/EDTA, followed by heating at 95 °C for 10 min achieved viral RNA extraction without commercial kits [21]. Both studies showed notably high clinical sensitivity and specificity (Table 1), demonstrating that rapid and cheap viral RNA extraction methods are suitable for RT-LAMP CRISPR-based SARS-CoV-2 detection methods.

In a study, RNA extraction on a chip using isotachophoresis (ITP) was demonstrated [22]. This study suggested that the ITP-based extraction of nucleic acid is compatible with downstream amplification by performing qPCR for the gene of interest (E gene) and a control gene (RNase P) as a validation assay. ITP is a basic electrophoretic separation technique that separates charged components in an electric field owing to the variation in their electrophoretic mobilities. This approach does not require sample preparation and is quick and applicable to a wide range of samples [23]. On the other hand, rather than extracting RNA from patients’ samples, a study utilized a lysis technique using the QuickExtract DNA Extraction kit (Lucigen, Middleton, WI, USA) that consists of a simple vortex-mixing step to break down viruses in order to release viral RNA [17]. The kit contains detergents and proteinase K, both of which can inactivate viral particles. The authors also suggested adding Proteinase K Inhibitor working solution if the samples are heated at 60 °C (10 min) instead of 95 °C to inactivate proteinase K before proceeding with the amplification step. 

### 2.2. RNA Amplification

**Figure 1:**
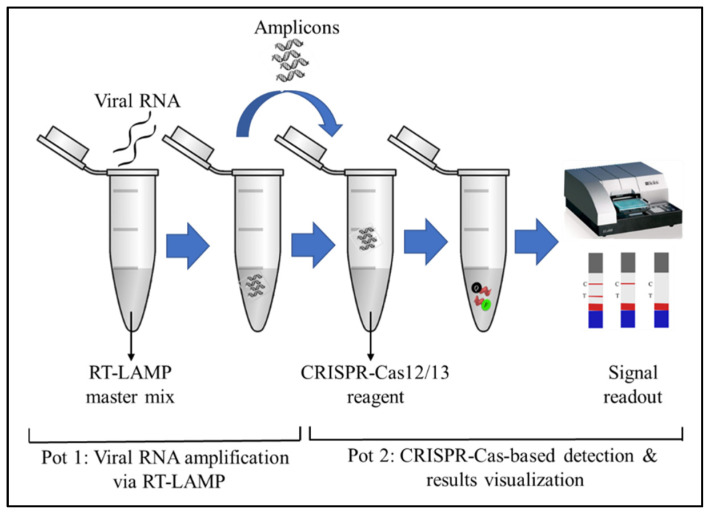
Schematic illustration showing a two-pot reaction for the detection of SARS-CoV-2. The viral RNAs are amplified via RT-LAMP in the first pot. The amplicons are then used for the CRISPR-Cas12/13-based detection, followed by the visualization of the result through colorimetry or a fluorescence assay in the second pot.

After viral RNA isolation, an amplification step was adopted by performing RT-LAMP to improve the sensitivity of the assay. RT followed by CRISPR-Cas-mediated detection was insufficient to detect viral RNA in samples with low SARS-CoV-2 viral loads [17], highlighting the importance of performing additional amplification before CRISPR-Cas detection. RT-LAMP was used to convert viral RNA into complementary DNA (cDNA), which was then amplified using a constant (60–65 °C) temperature. RT-LAMP does not require the use of a thermocycler, and the reaction time is shorter than that for the RT-qPCR approach. Furthermore, LAMP reagents are more widely available from various commercial sources, and the LAMP buffers are well-defined and can be systematically optimized with the Cas enzyme [17]. However, for the detection of SARS-CoV-2 RNA, RT-LAMP is performed through two sequential reactions: (1) the amplification of the viral RNA via RT-LAMP is performed; (2) a Cas endonuclease is used to detect the resultant amplicons, as illustrated in Figure 1 [24,25]. In the two-pot reaction, the opening of tubes is required after RT-LAMP, increasing the risk of amplicon contamination. The amount of target nucleic acid in the tube following the RT-LAMP reaction was high. When the tubes are opened, the amplicons may generate aerosol contamination, which can lead to high false-positive rates. Thus, RT-LAMP-based detection is not permitted outside well-controlled laboratory settings [26]. As a result, various research has been performed to develop a one-pot approach to POC testing that simplifies operations and eliminates the contamination risk in the detection of SARS-CoV-2.

**Figure 2:**
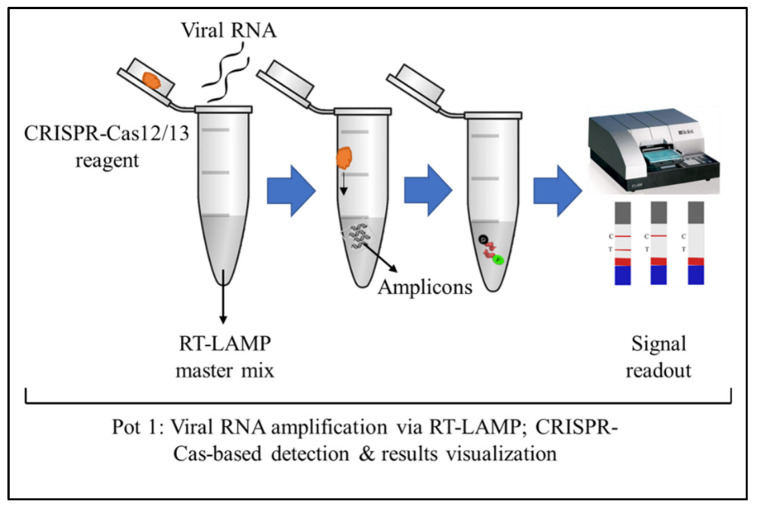
Schematic illustration shows the one-pot reaction for detecting SARS-CoV-2. The viral RNA amplification via RT-LAMP, CRISPR-Cas12/13-based detection, and visualization of the results through the fluorescence assay, all performed in a single tube. Alternatively, the tube can be opened for a lateral flow assay.

In a study, the authors developed an RT-LAMP and Cas12a-based one-pot assay to detect SARS-CoV-2 [8]. The RT-LAMP solution was added to the bottom of the tube and covered with mineral oil to avoid the volatilization of the reaction solution and amplicon contamination, to prevent false positive results in the subsequent detections. CRISPR-Cas12a reaction solution was added to the inner wall of the tube lid before the RT-LAMP reaction occurred. The CRISPR-Cas12a reagents did not drip because of the surface tension. When the RT-LAMP reaction was finished, the tube was shaken to mix the Cas12a-based detection reagents with the RT-LAMP amplicons. This method eliminated the potential aerosol contamination generated by opening the lid. However, by adding the CRISPR-Cas12 reagent as a droplet, it can be problematic since the CRISPR reagents can evaporate during the amplification process. Furthermore, the droplet may mix with the extracted viral RNA. This study may not be applicable to large-scale POC test. Another study had overcome the above-mentioned issue by using prepared PCR tubes with dried reagent mixtures. These PCR tubes are ready to be transported and use for on-site detection. These tubes were also successfully used for SARS-CoV-2 detection after the reagents were rehydrated. As demonstrated in Figure 2, the one-pot reaction require less steps and is ideal for POC use [27].

**Table 1:** Summary of Cas12- and Cas13-based detection of SARS-CoV-2 nucleic acid using RT-LAMP as an amplification method.

Name of the Method	Cas Enzyme	Target Region	Type of Clinical Samples	Number of Steps	Readout Method	Instrument Requirement	Assay Time *	Limit of Detection	Number of Clinical Samples	Sensitivity and Specificity (%)	ASSURED Criteria	Ref.
opvCRISPR	Cas12a	S	Nasopharyngeal swab	One	Fluorescence	Blue light	45 min	5 copies	50	100 and 100	Yes	[7]
iSCAN	Cas12a and Cas12b	N, E	Nasopharyngeal swab	One or two	Fluorescence or LFA	Fluorescence reader	60 min	10 copies/reaction	24	86 and 100	Yes (LFA)	[4]
DETECTR	Cas12a	N, E	Nasopharyngeal swab	Two	Fluorescence or LFA	Fluorescence reader	45 min	10 copies/µL	82	95 and 100	Yes (LFA)	[24]
-	Cas12a	ORF1ab	Respiratory swab	One	Fluorescence	Smartphone and 3D printing instrument	45 min	20 copies/reaction	10	100 and 100	Yes	[8]
CRISPR-ENHANCE	Cas12a with 3′DNA7-modified crRNA	N	-	Two	Fluorescence or LFA	Fluorescence reader	40 min	3–300 copies	-	-	Yes (LFA)	[25]
DETECTR	Cas12a	N	Nasopharyngeal swab, bronchoalveolar lavage, sputum	Two	Fluorescence or LFA	Fluorescence reader	30 min	50 copies	378	93 and 95.5	Yes (LFA)	[6]
ITP-CRISPR	Cas12a	N, E	Nasopharyngeal swab	One	Fluorescence	Inverted epifluorescence microscope	30 min	10 copies/µL	8	75 and 100	No ^a^	[22]
VaNGuard	Cas12a	S	Nasopharyngeal swab	Two	Fluorescence or LFA	Fluorescence reader	30 min	93 copies/reaction	-	-	Yes (LFA)	[28]
-	Cas12a	N, E	Respiratory swab	One	Fluorescence	Handheld UV lamp	40 min	N-30 E-45 copies/µL	100	94 and 100	Yes	[27]
STOPCovid	Cas12b	N	Nasopharyngeal swab, saliva	One	Fluorescence or LFA	Fluorescence reader	40–70 min	100 copies/reaction	17	91.7 and 100	Yes (LFA)	[17]
STOPCovid.v2	Cas12b	N	Nasopharyngeal swab, anterior nasal swab	One	Fluorescence	Fluorescence reader	45 min	0.033 copies/µL	402	93.1 and 98.5	No ^a^	[29]
-	Cas12a	N	Nasal swab	Two	Fluorescence	Blue-light transilluminator	40 min	16 copies/µL	12	100 and 100	No ^a^	[20]
RCSMS	Cas12a	E	Saliva	Two	Fluorescence and LFA	Fluorescence reader	40 min	5 copies/reaction	276	93.8 and 99	Yes (LFA)	[21]
CLAP	Cas12a	N	-	Two	Colorimetry	-	40 min	4 copies/µL	-	-	Yes	[30]
-	Cas12a	N, E	Nasopharyngeal swab	Two	Colorimetry	-	45 min	225 copies/µL	54	92.6 and 100	Yes	[31]
WS-CRISPR	Cas12a	N	-	One	Fluorescence	LED blue light or UV light	90 min	50 copies/μL	32	-	No ^b^	[32]
dWS-CRISPR			Nasal swabs and saliva					5 copies/μL			No ^b^	
Sherlock^TM^ CRISPR SARS-CoV-2	Cas13a	ORF1ab & N	Nasopharyngeal swab	Two	Fluorescence	Fluorescence reader	1 h	ORF1ab-6.75 N-1.35 copies/µL	60	100 and 100	No ^a^	[33]
DISCoVER	Cas13a	N	Nasal swab, saliva	Two	Fluorescence	Microfluidic cartridge, compact fluorescence reader	35 min	40 copies/μL	63	93.9 and 100	No ^a^	[34]

SARS-CoV-2: Severe acute respiratory syndrome coronavirus-2; RT-LAMP: Reverse transcription loop-mediated isothermal amplification; LFA: Lateral flow assay; UV: Ultraviolet; crRNA: CRISPR RNA; CRISPR: Clustered regularly interspaced short palindromic repeats. ^a^: required non-portable complex instruments for on-site testing; ^b^: required QuantStudio 3D digital chip. * Assay time excluding RNA extraction.

Changes in the pH and Mg^2+^ level during the RT-LAMP assay procedure were also a concern for the researchers when combining the RT-LAMP and CRISPR-based detection reactions in a single tube. RT-LAMP reduced the concentration of Mg^2+^ and pH of the reaction mixture to levels that were insufficient for Cas12a activity [35]. In order to compensate for the decreased Mg^2+^ level and pH drop during the RT-LAMP reaction, 40 mM Mg^2+^ and 50 mM Tris-HCl buffer were included in the Cas12a reagent mixture [27]. Another study showed that Cas12a from Recombinant *Acidaminococcus* sp. BV3L6 (A.s. Cas12a) has relatively high collateral cleavage activity at a low concentration of Mg^2+^ (2 mM). During isothermal amplification, DNA polymerase continuously consumes dNTPs (deoxyribonucleotide triphosphate) and produces a large number of pyrophosphate ions that can chelate Mg^2+^ to form magnesium pyrophosphate precipitate as the reaction byproduct. Thus, pyrophosphatase (PPase) is added into the reaction system to degrade the magnesium pyrophosphate precipitate and release free Mg^2+^, maintaining a constant Mg^2+^ concentration [32].

The optimal temperature and time for RT-LAMP have been determined: (i) temperature: 65 °C; (ii) time: 15 min [28]. However, most Cas12a nucleases have optimal activity at 37 °C. Therefore, phosphorothioated inner primers are beneficial for developing low-temperature isothermal amplification (52 °C) with Bst DNA polymerase [32]. An experiment showed that Bst 3.0 DNA polymerase significantly improved the amplification efficiency of RT-LAMP [30].

The RT-LAMP products were analyzed by gel electrophoresis at 120 V for 30 min. The bands represented the presence of amplicons after RT-LAMP by comparing to non-template control (NTC). This finding can also confirm that the primers do not generate non-specific amplification [7,30]. The RT-LAMP product from the one-pot assay was examined using gel electrophoresis, and a weak amplification of the target viral RNA was identified. This finding led to the hypothesis that having the active Cas12b–crRNA complex in the same pot causes digestion of the initial RT-LAMP product, which has a major impact on the RT-LAMP amplification performance and, hence, the detection robustness [4]. 

### 2.3. Cas12-Based Detection

**Figure 3:**
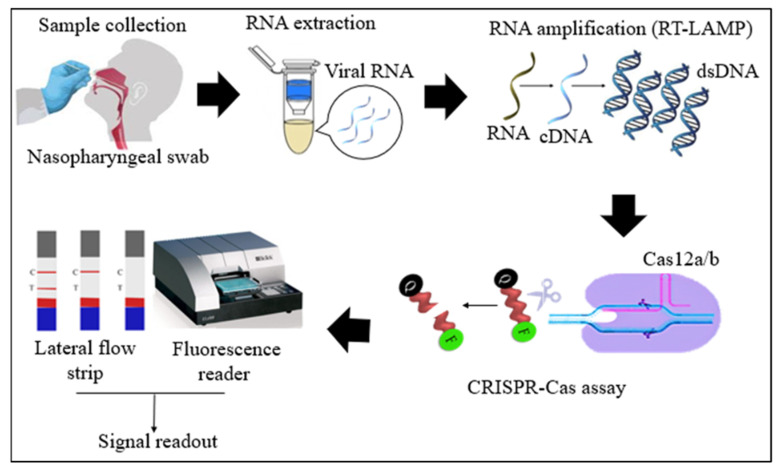
Schematic diagram of RT-LAMP CRISPR-Cas12a/b-based detection of SARS-CoV-2. Samples (nasopharyngeal swabs) were collected from symptomatic and asymptomatic individuals, and viral RNAs were extracted. With the RT-LAMP step, viral RNAs are first converted into cDNAs, which are subsequently amplified. The amplicons were targeted in CRISPR-Cas-based detection, and the results of the tests were visualized via colorimetry or a fluorescence assay.

Cas12 proteins are effector nucleases of the Class 2 type V CRISPR-Cas systems. Cas12 enzyme performs two types of cleavage activity such as cis-cleavage (specific) activity against dsDNA targets and trans-cleavage (collateral or non-specific) activity on ssDNA non-targeted sequences. The collateral activity is the foundation for very specific and sensitive nucleic acid detection methods. In COVID-19 diagnosis, Cas12 endonucleases can randomly cleave the non-target DNA (reporter probe) once activated by a single- or double-stranded DNA target sequence complementary to their crRNA [4,36]. Two orthologs of the Cas12 family, Cas12a, and Cas12b, have been widely used to detect SARS-CoV-2 nucleic acid. Cas12b is significantly smaller and more thermostable than Cas12a [37,38]. In the RT-LAMP and Cas12-based SARS-CoV-2 RNA detection assays, the RT-LAMP amplicons are introduced to the Cas12/crRNA complex. After the specific binding of crRNA to the target DNA amplicon, Cas12 nuclease performs collateral cleavage on the non-target reporters, as shown in Figure 3 [4,24]. The fluorescence reporter probe is made up of a short ssDNA with a fluorophore on one end and a quencher on the other. Fluorescence is suppressed until Cas12 performs collateral cleavage and degrades the reporter probe. The fluorophore is then liberated, resulting in the production of a fluorescent signal.

As LAMP (60–65 °C) operates at a higher temperature than RPA (37–42 °C), an RT-LAMP, and CRISPR-based one-pot SARS-CoV-2 RNA detection assay demands a Cas enzyme with thermostable collateral activity. A study proved that the Cas12b variant from *Alicyclobacillus acidiphilus* (AapCas12b) exhibited adequate activity in the same temperature range as LAMP [39]. However, there are no published crRNA design criteria for AapCas12b. According to a study, reactions involving the AapCas12b enzyme with AacCas12b sgRNA yield more intense and selective nuclease activity, and the addition of taurine enhanced the thermal stability of the Cas12b enzyme in a one-pot reaction [17]. It was also discovered that 3′-DNA with 7-mer extensions on the crRNA significantly improved the trans-cleavage ability of activated Cas12a [25]. On the other hand, the phosphorothioate ssDNA extensions at the 3′ or 5′ end showed limited or no activity, implying that extending the crRNA with 13-mer phosphorothioate ssDNA suppresses Cas12a trans-cleavage activity. The content of the reporter probe is also important for sensitivity, as a 12-nucleotide probe yielded a higher signal-to-noise ratio than an eight-nucleotide probe [6], and the use of two crRNAs targeting distinct sites of the SARS-CoV-2 genome prevents false-negative results. 

In a study, the CRISPR-mediated detection step was optimized to prevent false-negative results caused by SARS-CoV-2 genome alterations [28]. Cas12a from the *Lachnospiraceae bacterium* (LbCas12a) was combined with two distinct crRNAs to target the RT-LAMP amplicons. When one of the target sites is mutated, the other crRNA can still function properly, resulting in positive COVID-19 results. Another study developed the CLAP (Cas12a-assisted RT-LAMP/AuNP) assay by utilizing gold nanoparticle (AuNP) probes modified with different DNA (DNA1 and DNA2) that could be crosslinked by a linker-ssDNA, resulting in a color change. In the presence of the viral RNA, the trans-cleavage activity of the Cas12a enzyme could be activated, resulting in the cleavage of linker-ssDNA, with the color remaining red. In the absence of viral RNA, the linker-ssDNA hybridizes with AuNP-DNAs and the color changes from red to purple [30].

### 2.4. Cas13-Based Detection 

**Figure 4:**
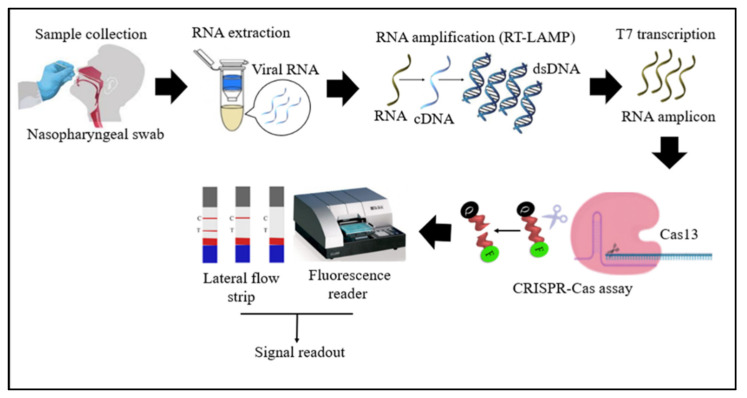
Schematic diagram of RT-LAMP CRISPR-Cas13-based detection of SARS-CoV-2. Samples (nasopharyngeal swabs) were collected from symptomatic and asymptomatic individuals, and viral RNAs were extracted. With the RT-LAMP step, viral RNAs are first converted into cDNAs, which are subsequently amplified. An additional step was required for Cas13-based detection: T7 transcription to convert DNA amplicons to RNA amplicons, which were then targeted in Cas13-based detection; the results of the test were visualized via colorimetry or a fluorescence assay.

**Figure 5:**
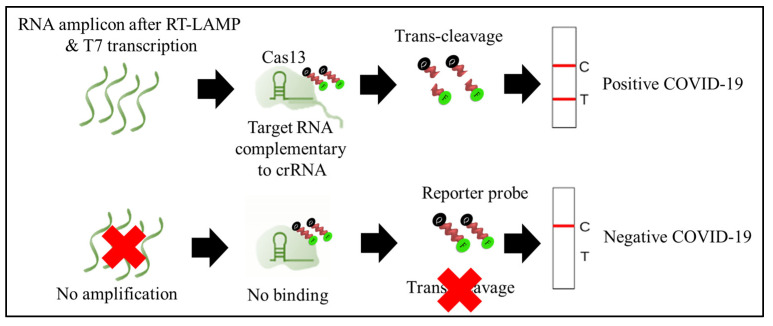
Schematic diagram depicted the trans-cleavage activity of Cas13 enzyme. In the presence of SAR-CoV-2 virus, the RNA amplicon after RT-LAMP and T7 transcription was complemented to pre-designed crRNA (ribonucleoprotein complex, Cas13 + crRNA + amplicon). As a result, the Cas13 enzyme was activated to cleave the reporter probes, and the lateral flow assay (IFA) showed both a control (C) and a test (T) line, indicating a positive COVID-19 result. In absence of SAR-CoV-2 virus, there was no amplification of target site and binding to crRNA. Thus, Cas13 enzyme remain inactivated, and the reporter probes were not cleaved. The LFA showed only control line, indicating negative COVID-19 result. The un-cleaved reporter molecules are captured at the first detection line (control line), whereas indiscriminate Cas13 cleavage activity generates a signal at the second detection line (test line).

Cas13 proteins are effector nucleases of the Class 2 type VI CRISPR-Cas systems. Unlike other Cas nucleases of Class 2 CRISPR-Cas systems, Cas13 targets single-stranded RNA (ssRNA) instead of DNA [4]. Cas13 also demonstrates collateral RNA cleavage activity when activated by a target RNA complementary to its crRNA. The stem-loop structure of the crRNA is critical for ssRNA cleavage. Therefore, Cas13a crRNA constructed with a single stem-loop specific to Cas13a and a protospacer domain-specific to the target [12]. The crRNAs must have minimal sequence overlap with the primer sequences; thus, a comparative in-silico analysis of RT-LAMP primers and guide RNAs with the targeted viral regions should be performed to avoid detection of non-specific amplification products by Cas13 to ensure specificity of assay [34]. As Cas13a proteins are triggered only by RNA targets, an additional T7 transcription step is required to convert the DNA amplicons to RNAs after the RT-LAMP reaction (Figure 4) [33]. In addition, as triggered Cas13a cleaves ssRNA rather than ssDNA (Figure 5), the reporter probe for the Cas13a system should be introduced as an ssRNA rather than ssDNA. Compared to Cas12, Cas13 enzymes have been used less frequently in RT-LAMP CRISPR-based SARS-CoV-2 detection studies because Cas13 requires an extra T7 transcription step and operates at lower temperatures than those at which RT-LAMP occurs. The DISCoVER (Diagnostics with Coronavirus Enzymatic Reporting) assay is an RNA extraction-free test that combines the RT-LAMP amplification method with a Cas13-mediated probe. This assay utilizes LbuCas13a (*Leptotrichia buccalis* Cas13a) because it is significantly faster than LbCas12a (Lachnospiraceae bacterium ND2006 Cas12a) at low concentrations of the activator. In addition, the assay is carried out in a portable microfluidic device with real-time fluorescent detection [34].

### 2.5. Signal Readout

Various readout mechanisms, especially fluorescence [8] and colorimetric [24] methods, have been developed for CRISPR-based assays to detect SARS-CoV-2. However, fluorescence signal measurement requires special instruments, such as a fluorescence reader, inverted epifluorescence microscope, handheld UV lamp, or 3D printing device, which are typically large, costly, and unsuitable for POC applications [4,8,22]. On the other hand, colorimetric assays are ideal for POC applications because these approaches are simple to use, inexpensive, and readily available. The lateral flow assay (LFA) seems to be the most popular colorimetric readout technique [24]. The LFA also enables POC testing solutions that can be used in areas where SARS-CoV-2 infection is most likely to spread—airports, public hospitals, and regional medical centers—especially in countries with limited resources. The LFA is appropriate for large-scale testing for the early diagnosis of SARS-CoV-2 carriers, enabling them to be efficiently isolated and quarantined, hence minimizing the transmission of the virus. Apart from that, a microfluidic method was developed in a study, which uses a minimal amount of reagents on the chip and can automatically detect RT-LAMP-amplified cDNA by employing ITP-mediated CRISPR-Cas12 DNA detection [22]. Another study showed the reaction mixture (RT-LAMP and CRISPR-Cas12a) distributed into a QuantStudio 3D digital chip (Thermo Fisher Scientific, Waltham, MA, USA), with the micro-reactions with target RNA showing strong green fluorescence (positive spots), while those without targets did not (negative spots) [32].

### 2.6. Genes of Interest and Assay Time

The genome of SARS-CoV-2 comprises open reading frame 1ab (ORF1ab), encoding the ORF1ab polyprotein (two-third), and genes encoding structural proteins (one-third): the spike (S), envelope (E), membrane (M), and nucleocapsid (N) proteins [40]. Several studies have been conducted using the RT-LAMP and CRISPR-based detection of SARS-CoV-2 targeting various regions of the viral RNA: the S, E, N, and ORF1ab sequences. The regions N and E have been used more often than S and ORF1ab. Researchers are concentrating their efforts on the S gene, which encodes a spike protein that enables SAR-CoV-2 to enter host cells [41]. In a Cas12a-based SARS-CoV-2 detection study, the human RNase P gene was employed as a positive control for the viral RNA extraction and Cas12a-mediated detection steps [24]. The time required to complete the overall process, from RNA extraction to result interpretation, is generally between 30 and 45 min. Moreover, CRISPR-based SARS-CoV-2 detection methods allow for on-site detection, which further reduces the time to obtain results compared to the gold standard approach, RT-qPCR, which requires collecting samples to be transferred to a centralized laboratory. 

### 2.7. Limit of Detection (LOD), Sensitivity, and Specificity

The LOD is defined as the lowest number of viral RNA copies that can be reliably detected with a given sample. The LODs for the RT-LAMP CRISPR-based detection of SARS-CoV-2 differ markedly between methods (Table 1). The lowest LOD (0.033 copies/µL) was achieved by performing the magnetic-bead-mediated concentration of SARS-CoV-2 RNAs, meaning that the LOD of STOPCovid.v2 was 30-times lower than that of the RT-qPCR test approved by the CDC [29]. This study indicates that the optimization of RT-LAMP CRISPR-based detection enables sensitivity superior to that of RT-qPCR in diagnosing COVID-19. 

Sensitivity is the capacity of a test to correctly identify an individual with a disease. By contrast, specificity is the ability of a test to accurately identify persons who do not have the disease [42]. The clinical sensitivity and specificity of the RT-LAMP CRISPR-based SARS-CoV-2 detection methods were in the ranges of 75–100% and 95.5–100%, respectively (Table 1). Several strategies for improving the sensitivity and specificity of RT-LAMP CRISPR-based SARS-CoV-2 detection have been explored, such as the utilization of multiple crRNAs, optimization of the RT-LAMP temperature and reaction time [28,43], modification of the crRNA [25], optimization of the length of the reporter probe [6], and addition of bovine serum albumin and L-proline to Cas12/Cas13-based detection reactions [44].

## 3. Conclusions

The use of RT-LAMP and CRISPR technologies in the detection of SAR-CoV-2 is a novel technique that has made significant progress and has the potential to be further developed. In most CRISPR-Cas-mediated SARS-CoV-2 detection methods, Cas12 or Cas13 proteins have been used as the CRISPR effectors. Patients might be able to obtain their COVID-19 testing results in less than an hour at a reasonable cost if this technique becomes available in hospital settings, which would become a vital tool during this disease outbreak. With these methods, on-site screening could be applied more widely to prevent asymptomatic carriers from spreading the infection to others unintentionally. Following the necessary improvements in the RT-LAMP CRISPR-based detection with a combination of LFA, it is expected to be offered in healthcare environments or as a diagnostic kit for use at home possibly in the future. To date, the FDA has only approved two CRISPR-based COVID-19 diagnostic tools for emergency use: SHERLOCK and DETECTR. Both are still two-step methods excluding RNA extraction, and SHERLOCK evaluates the data using a fluorescence reader, which is less effective for a POC test. Our recommendations for RT-LAMP CRISPR-mediated SARS-CoV-2 detection are (i) applying a rapid RNA extraction method or simplifying the RNA-extraction step followed by RT-LAMP amplification and CRISPR-mediated detection in one pot; (ii) performing CRISPR-Cas-based detection using thermostable Cas12b with double crRNA and optimized buffer conditions; and (iii) finally, visualizing the assay results using LFA. These optimizations will make CRISPR-based COVID-19 diagnostic tests more sensitive and easier to use and will further facilitate the applicability of these tests at the POC.

## Data Availability

Not applicable.
